# Generation, Characterization and Application of Antibodies Directed against HERV-H Gag Protein in Colorectal Samples

**DOI:** 10.1371/journal.pone.0153349

**Published:** 2016-04-27

**Authors:** Christina S. Mullins, Maja Hühns, Mathias Krohn, Sven Peters, Valérie Cheynet, Guy Oriol, Michèle Guillotte, Sandrine Ducrot, François Mallet, Michael Linnebacher

**Affiliations:** 1 University Medicine Rostock, Department of General Surgery, Molecular Oncology and Immunotherapy, Schillingallee 69, 18057 Rostock, Germany; 2 University Medicine Rostock, Institute of Pathology, Strempelstraße 14, 18055 Rostock, Germany; 3 Joint Unit Hospices Civils de Lyon, bioMérieux, Cancer Biomarkers Research Group, Centre Hospitalier Lyon Sud, Bâtiment 3F, 69495, Pierre Bénite cedex, Lyon, France; 4 Global R&D Immunoassay, bioMérieux, Marcy l’Etoile, France; 5 R&D Immunoassay, bioMérieux, Raw Material Department, Marcy l’Etoile, France; 6 EA Pathophysiology of injury-induced immunosuppression, University of Lyon1–Hospices Civils de Lyon–bioMérieux,Hôpital Edouard Herriot, 5, Place d’Arsonval, 69437 LYON Cedex 3, Lyon, France; Plymouth University, UNITED KINGDOM

## Abstract

**Introduction:**

A substantial part of the human genome originates from transposable elements, remnants of ancient retroviral infections. Roughly 8% of the human genome consists of about 400,000 LTR elements including human endogenous retrovirus (HERV) sequences. Mainly, the interplay between epigenetic and post-transcriptional mechanisms is thought to silence HERV expression in most physiological contexts. Interestingly, aberrant reactivation of several HERV-H loci appears specific to colorectal carcinoma (CRC).

**Results:**

The expression of HERV-H Gag proteins (Gag-H) was assessed using novel monoclonal mouse anti Gag-H antibodies. In a flow cytometry screen four antibody clones were tested on a panel of primary CRC cell lines and the most well performing ones were subsequently validated in western blot analysis. Finally, Gag-H protein expression was analyzed by immune histology on cell line cytospins and on clinical samples. There, we found a heterogeneous staining pattern with no background staining of endothelial, stromal and infiltrating immune cells but diffuse staining of the cytoplasm for positive tumor and normal crypt cells of the colonic epithelium.

**Conclusion:**

Taken together, the Gag-H antibody clone(s) present a valuable tool for staining of cells with colonic origin and thus form the basis for future more detailed investigations. The observed Gag-H protein staining in colonic epithelium crypt cells demands profound analyses of a potential role for Gag-H in the normal physiology of the human gut.

## Introduction

Human endogenous retroviruses (HERV) are relics of retroviral infections. About 200,000 copies in the human genome, roughly making up 5% of the chromatin, are arranged in at least 31 families [1,2]. To date, all HERV elements that have been characterized are defective for viral replication and it is generally accepted that HERVs are silent due to mutation and epigenetic regulation [3]. Their structure consists of the genes gag, pol and env flanked by long terminal repeats at their 5’ and 3’ ends [4]. Gag proteins are the primary retroviral structural proteins concocting the viral core [5,6]. More precisely, Gag proteins mediate the intracellular transport to the cell membrane, direct assembly and facilitate budding of the viral particles [5]. The protein itself is generally localized in the cytoplasm [7] and is sufficient for the formation of virus-like particles [8].

Permissive HERV reactivations are often associated with pathological contexts including cancer [9]. For example, transcripts and proteins originating from HERV-K HML-2 loci have been associated with melanoma [10], leukemia and lymphoma [11,12] as well as tumors of the breast [11,13] and ovary [14]. Further, expression of the HERV-E family has been associated with prostate cancer [15], individual loci from the HERV-W group were found reactivated in seminoma [16] and transcripts from the HERV-R family were detected in liver and lung cancers [9]. Strikingly, expression of several HERV-H loci has been described to be colorectal cancer (CRC) specific [17–21].

CRC remains the second cause of cancer-related deaths in Europe and the United States. Its occurrence is closely connected to genetic background, chronic inflammation, lifestyle and dietary habits [22]. When looking at the general mutation level, specific mutations and other molecular changes, at least three molecular subtypes of CRC are currently well recognized: (I) chromosomal instable tumors (these are microsatellite stable (MSS)), (II) microsatellite instable (MSI) tumors and (III) tumors presenting with the CpG island methylation phenotype [23].

### Aim of the study

HERV-H expression analyses so far have been restricted to the RNA level. For HERV-H protein analyses (on CRC cells) HERV-H family specific anti-Gag mouse monoclonal antibodies were generated. Analyses of the obtained clones included their basic characterization, screening of HERV-H Gag protein (Gag-H) expression on patient derived low-passage CRC cell lines, selection of most well performing clone(s) and staining of CRC patient tissue (tumor and normal adjacent colon tissue pairs). This finally provided a more detailed picture of the subcellular localization of Gag-H proteins in colonic cells.

All in all, we could address the question if, where and to which extent HERV-H Gag proteins are expressed in tumoral and normal colon tissues. The novel Gag-H antibody clone(s) described here are effective for assessment of HERV-H protein expression in CRC tumor models as well as patient samples and represent a valuable tool for subsequent analyses.

## Material and Methods

All procedures were approved by the Ethics Committee of the Medical Faculty at the University of Rostock (Ethikkommission an der Medizinischen Fakultät der Universität Rostock, St.-Georg-Str. 108, 18055 Rostock, Germany; reference number II HV 43/2004) in accordance with guidelines for the use of human material. An informed consent form was obtained in writing for all patients.

### Immunization with recombinant protein

The study was carried out in accordance with the recommendations of The Canadian Council on Animal Care in Science. The protocol was approved by the French Ministry of Higher Education and Research (Permit Number 01004.01) and all efforts were made to minimize animal suffering. For the immunization, 3 Balb/C female mice were used. They were provided by Charles River Laboratories. Animals were kept in standard cages with 14h light and 10h dark cycles, and free access to water as well as pellets. Mice were routinely checked twice a week. Finally, animals were euthanized by CO_2_ inhalation.

**Fig 1:**
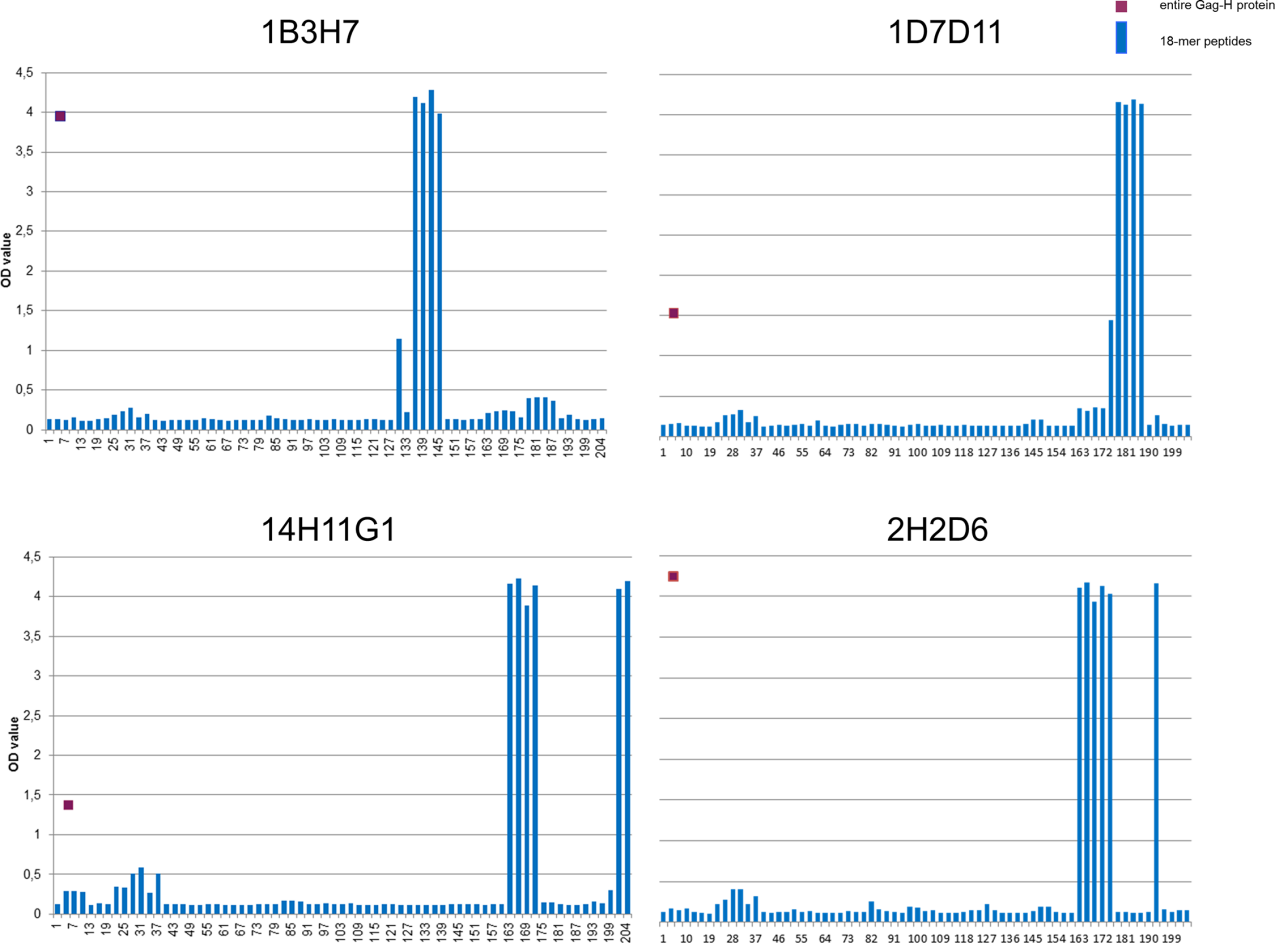
Epitope mapping. Representative bar charts for epitope mapping are depicted. Upper left: clone 1B3H7, upper right: clone 1D7D11, lower left: clone 14H11G1 and lower right: clone 2H2D6.

The HERV-H Gag sequence (on chromosome 3, 46368229–46373908 (assembly 37/19)) was PCR amplified ((I) outer: forward primer (616R):5’-GCA GGA GGT TAG TGT CAG-3’ and reverse primer (617R): 5’-GGT TAA TTT GCA GAC ACT AAC-3’; (II) inner: forward primer (623R): 5’-CCA TGG GCA GCC TTC CAC C-3’ and reverse primer (624R): 5’-AAG CTT ACT AAT TTG GGA GAG GTC AGA TAA AGT AAA-3’) from the genomic DNA of a healthy female volunteer. The Gag-H PCR product was inserted into the pCRII vector and from there cloned Nco1 / Xba1 into the pGEX-3X expression vector. Recombinant HIS tagged GST-Gag-H protein was expressed in E. coli BL21, isolated and purified. Mice were inoculated with 50μg recombinant GST-Gag-H protein (for full amino acid sequence see Fig 1) at days 0, 14, 28 and 102 with Freund's complete adjuvant, on days 103, 104, 105 and 106 mice were boosted with 100μg recombinant Gag-H protein and Freund's incomplete adjuvant. Mice were sacrificed, spleens were collected and sera were affinity purified. Splenocytes were fused with myeloma cells; obtained hybridoma cells were seeded, selected in HAT medium and subsequently cloned. Produced antibodies were affinity purified on a protein A column using an AKTA chromatography system (GE Healthcare, Velizy-Villacoublay, France).

### ELISA

Specific HERV-H Gag protein reactivity of monoclonal Gag-H antibodies was assessed by coating ELISA plates with (I) the recombinant Gag-H protein used for inoculation of mice, (II) an irrelevant Gag-H fusion protein (GST-RRM1), (III) the GST protein alone, (IV) HERV-E (matrix), (V) HERV-K HML-2 (matrix), (VI) HERV-K HML-2 (capsid), (VII) HERV-K HML-5 (matrix), (VIII) HERV-W (matrix), (VIIII) HERV-W (capsid). Further, the following antibodies were used to verify recombinant protein integrity: anti-His (Qiagen, Venlo, Netherlands), anti-GST clone B-14 (Santa Cruz Biotechnology, Dallas, USA), anti-HERV-E matrix clone 9H6F6, anti-HERV-K HML-2 matrix clone 1B2G10, anti-HERV-K HML-2 capsid clone 4B12A8, anti-HERV-K HML-5 matrix clone 4H12B7, anti-HERV-W matrix clone 2G5E12, anti-HERV-W capsid clone 1C2F8. If not stated otherwise, proteins and antibodies were from bioMérieux (Marcy l’Etoile, France).

### Epitope mapping

A biotinylated peptide library was synthesized using Fmoc chemistry and designed to cover the sequence of the recombinant Gag-H protein. The library consisted of 69 synthetic peptides with a length of 18 residues and an overlap of 15 amino acids. Ninety-six-well- maxisorb-plates were coated with Streptavidin (10μg/ml in PBS– 1h at 37°C) and blocked with PBS/BSA (overnight at room temperature). Biotinylated peptides (1μg/ml) were added and incubated (1h at 37°C). Purified monoclonal antibodies were added (1μg/ml in PBS– 90min at 37°C). Plates were incubated with secondary antibody (alkaline phosphatase conjugated goat anti-mouse– 90min at 37°C) before substrate was added (para-nitrophenylphosphate); reaction was blocked with 1M NaCl and plates were read at 405nm.

### Cell culture and B-LCL generation

HEK293 were obtained from CLS (Cell Lines Service GmbH, Eppelheim, Germany). CRC cell lines (HROC18, HROC24, HROC32, HROC40, HROC87 T0 M2, HROC113 cT0 M1, HROC126, HROC147 T0 M1, HROC147Met, HROBMC01, HHC6548 T1 M1) [24–26] and GBM cell lines (HROG04, HROG07 and HROG38) [27–28] were generated in our lab out of tumors from patients of the surgical clinic. All cell lines were cultured in DMEM/Ham’s F12 supplemented with 10% fetal calf serum and 2mm L-glutamine. Cells were incubated at 37°C in 5% CO_2_. All cell culture plastics were purchased at Greiner bio one (Frickenhausen, Germany) and culture media as well as supplements were obtained from Pan-Biotech (Aidenbach, Germany). Peripheral blood mononuclear cells were isolated from the blood of three healthy donors by Saccharose-Epichlorhydrin-Copolymer density centrifugation. They were immortalized by EBV to generate B-LCL as described before [24].

### Flow cytometry screen of cell lines

For cytoplasmic staining of cells, 5x10^5^ cells were fixed with 2% Formafix and subsequently treated with buffer P (0.1% saponin, 0.01M Hepes, 1% FCS in PBS) for 10min at room temperature to permeabilize the cell membrane. Cells were incubated with 1μg anti-Gag-H antibody or with equal amounts of irrelevant antibody (2G2B3, an anti-His antibody) for 30min at room temperature and washed with buffer P. The secondary fluorescent-labeled goat anti-mouse antibody (DakoCytomation, Glostrup, Denmark) was added in buffer P for an additional 30min incubation at room temperature. Cells were washed and resuspended in 2% Formafix at a final volume of 200μl. Cells handled the same way but without addition of primary antibody served as negative controls.

### Western Blot

Protein extracts from snap frozen cell pellets (3x10^6^ cells) were separated by SDS-PAGE, transferred onto PVDF membranes and were probed with antibodies against Gag-H (1μg; clones 14H11G1, 1B3H7 or 1D7D11), against an irrelevant epitope (1μg anti-His, clone: 2G2B3) as negative control and against beta-actin (1:5,000, Cell Signaling Technology, Danvers, USA) as loading control. Proteins of interest were detected with fluorescent goat anti-mouse (Gag-H and irrelevant antibodies) or anti-rabbit (beta-actin antibody) antibodies (1:5,000, LI-COR, Lincoln, USA) and visualized with the Odyssey imager (LI-COR).

### Cytospin preparation and immunohistochemistry

For cytospin preparations, 3x10^4^ cells per spot were centrifuged onto an object slide and fixed for 10min with ice cold acetone. Four μm sections were cut from FFPE blocks and transferred to an adhesive-coated glass slide system (Instrumedics Inc, Hackensack, NJ, USA).

Immunohistochemical staining was performed with an autostainer (EnVisionTM FLEX, DAKO) according to the manufactures standard protocol with 5μg/ml primary antibodies (anti-Gag-H clones 14H11G1 and 1B3H7) and irrelevant antibodies (anti-His, clone 2G2B3 and anti-HIV P24, clone 3D8C6E7). Diamonobenzidine was used as chromogen for all immunoreactions. Cytospins were treated in the same way without pre-treatment of the slides.

## Results

### Mouse monoclonal anti-Gag-H antibodies

Antibodies were generated against a large open-reading frame from a locus on chromosome 3 (46368229–46373908; assembly 37/19) by immunization of mice with a recombinant GST-Gag-H fusion protein (for entire amino acid (AA) sequence see S1 Fig). Seven clones were obtained and screened for Gag-H protein specificity by ELISA. Since the recombinant GST-Gag-H fusion protein contained two tags, GST at the N-terminus and His at the C-terminus, protein integrity could be tested using antibodies against the tags. Although the tag-specific signals obtained were lower than those observed for the control proteins harboring both tags (i.e. HERV-W (matrix) and GST protein alone), as well as five additional control HERV proteins harboring only the His tag, significant reactivity above background was found for the GST-Gag-H fusion protein (Table 1). Integrity of the HERV proteins used as controls could also be demonstrated by monoclonal antibodies specific for the respective proteins (Table 1).

**Table 1:** ELISA analyses.

protein	control antibodies			anti-Gag-H antibody clones			
	anti-His	anti-GST	protein specific	1B3H7	1D7D11	2H2D6	14H11G1
**HERV-E (matrix)**	4.010	0.155	3.141	0.136	0.143	0.130	0.129
**HERV-K HML-2 (matrix)**	3.948	0.142	3.183	0.140	0.145	0.144	0.176
**HERV-K HML-2 (capsid)**	4.187	0.135	3.143	0.140	0.133	0.131	0.148
**HERV-W (matrix) GST**	1.183	0.535	3.230	0.164	0.131	0.224	0.134
**HERV-W (capsid)**	4.193	0.154	3.020	0.131	0.186	0.126	0.157
**HERV-K HML-5 (matrix)**	3.242	0.136	3.500	0.131	0.980	0.127	0.130
**GST**	2.477	0.518	3.154	0.138	0.229	0.123	0.126
**Gag-H GST**	0.748	0.331	n.d.	3.951	1.531	4.237	1.373

The OD_405_ values in the control antibody columns give information on the integrity of the proteins used, which are named in the first column. Antibodies used (distinguished in control and anti-Gag-H clones) are given in the headlines. Underlined values in the GST column can be considered background since the tested proteins have no GST-tag. Integrity of all proteins used is demonstrated with values above background of the anti-His antibody, of the anti-GST antibody (against the proteins with GST tag only) and of the different antibodies directed against the HERV control proteins.

Cross reactivity of the anti-Gag-H antibody clones with other HERV family proteins was also tested. Generally, values were at background level with the exception of 1D7D11 against HERV-K HML-5 (matrix), thus, we can exclude cross reactivity with the exception of 1D7D11 for HERV-K HML-5 (matrix). n.d., not done.

Subsequently, the seven antibody clones were tested for specificity against the GST tag. Three antibody clones reactive to the GST tag were discarded (data not shown).

The remaining four antibody clones (1B3H7, 1D7D11, 2H2D6 and 14H 11G1) were further analyzed for cross-reactivity towards proteins of other HERV families (Table 1). Only one of the four anti-Gag-H antibody clones (1D7D11) cross-reacted with a matrix protein of HERV-K HML-5. However, all four antibody clones gave a strong signal towards the Gag-H fusion protein used for initial immunization.

All obtained anti-Gag-H antibodies were of the IgG1 kappa isotype (data not shown).

### Epitope mapping of the monoclonal anti-Gag-H antibodies

**Fig 2:**
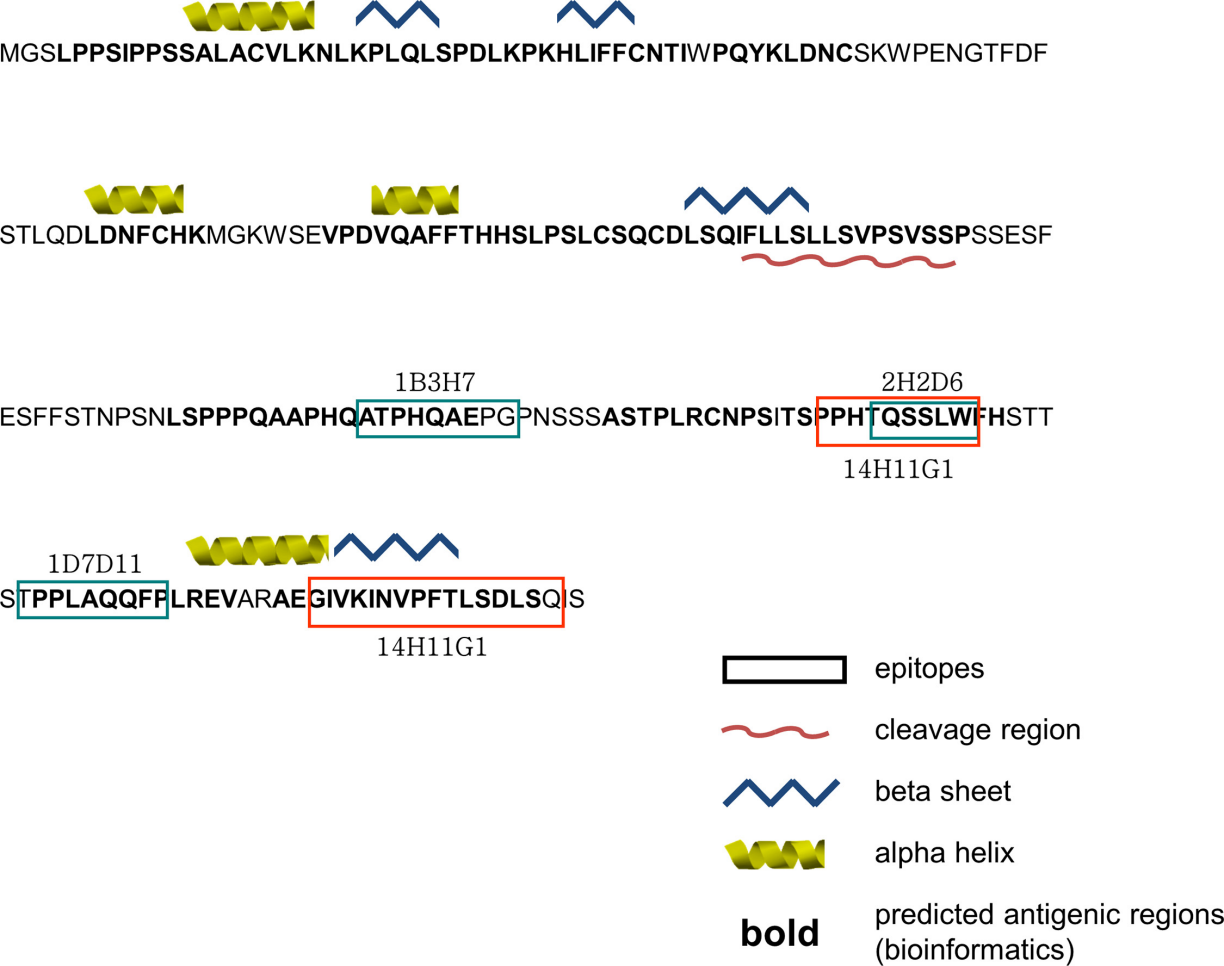
Gag-H protein sequence, structure and antibody epitopes. Protein features, predicted using Geneious 7.1.4 [37], are indicted as follows: alpha helix (yellow helix), beta sheet (blue zigzag), signal cleavage region (red wavy line) and antigenic region (bold). Epitopes were determined using a sequential overlapping peptide library. The minimal epitopes defined as the amino acid sequence shared by all peptides detected by the antibody are labeled in boxes and the names of monoclonal antibodies are indicated.

Epitope mapping was performed for the four Gag-H antibody clones using 18-mer peptides of the Gag-H protein with a 3-mer shift (Fig 1 and S1 Table). Four different epitopes were identified with the ones of clones 1B3H7, 1D7D11 as well as 2H2D6 being composed of single nine- or six-mers respectively; while the epitope for clone 14H11G1 seems more complex (composed of two epitope parts). One identified epitope region of 14H11G1 is highly similar to the six-mer epitope of clone 2H2D6 but with three additional AA at the N-terminus. Besides, a second 16-mer epitope with 57% homology to the first was identified located at the C-terminus of the protein for clone 14H11G1. The epitopes and their respective positions on the recombinant protein are shown in Fig 2.

In a bioinformatics homology screen of the HERV-H family, pan Gag-H reactivity was investigated for the antibody clones (S2 Table). Considering numerical coverage (% of epitope length) and AA identity conservation (% AA homology) only the epitope PPHTQSSLW (14H11G1) mapped to two genomic regions (on chr.3 and chr.13). However, when setting the AA identity to at least 80% for the short epitopes, up to 7 additional genomic locations were found (in case of the long 16-mer epitope 44 additional loci are found).

### Flow cytometry screen of CRC cell lines and western blot

**Fig 3:**
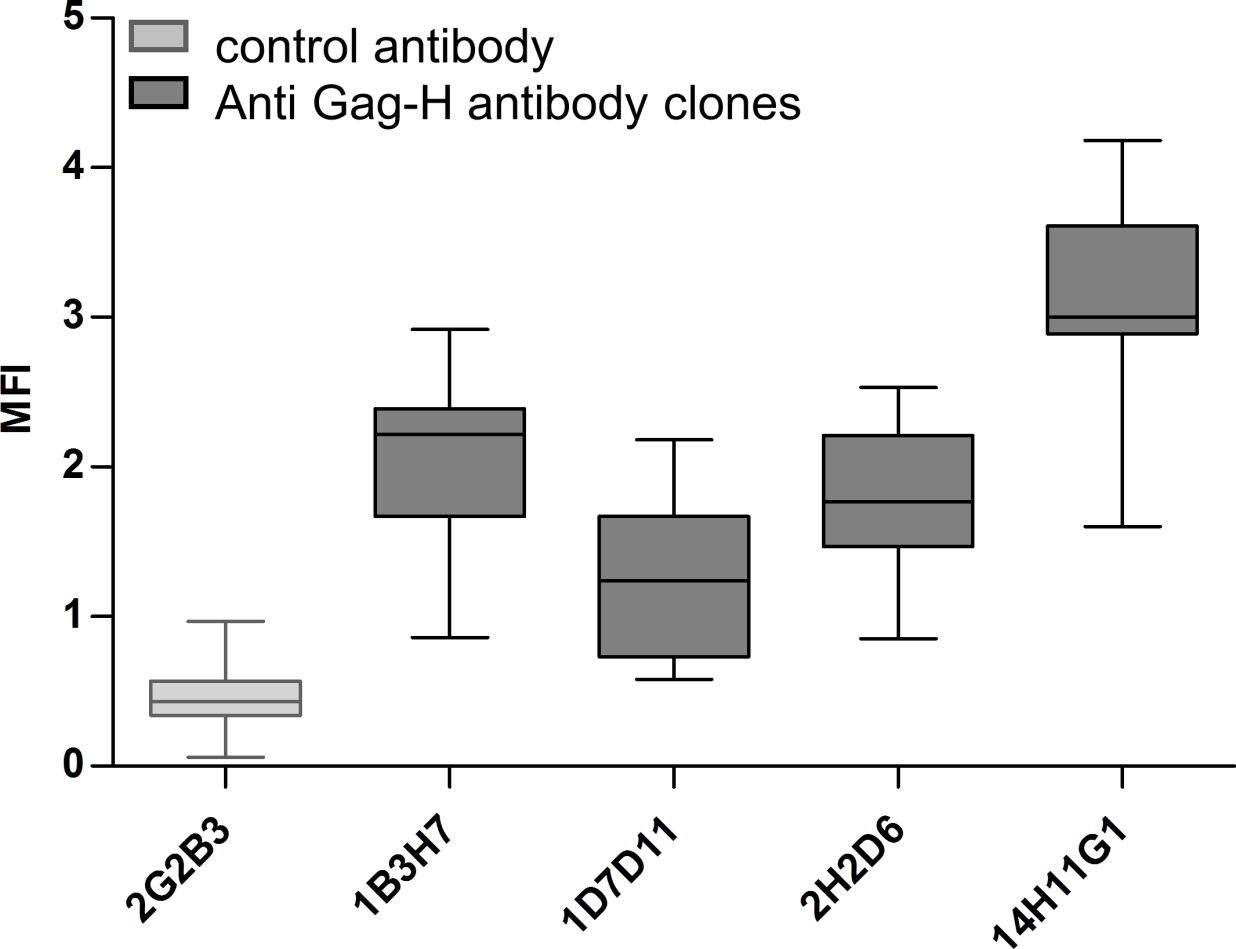
Gag-H protein expression in CRC cell lines. Expression of Gag-H proteins was assessed by flow cytometry. MFI for each antibody clone and CRC cell line is depicted in the box plot diagram. The irrelevant control antibody (anti-His, 2G2B3) is colored light grey whereas the different anti Gag-H antibody clones (1B3H7, 1D7D11, 2H2D6 and 14H11G1) are represented in dark grey.

**Fig 4:**
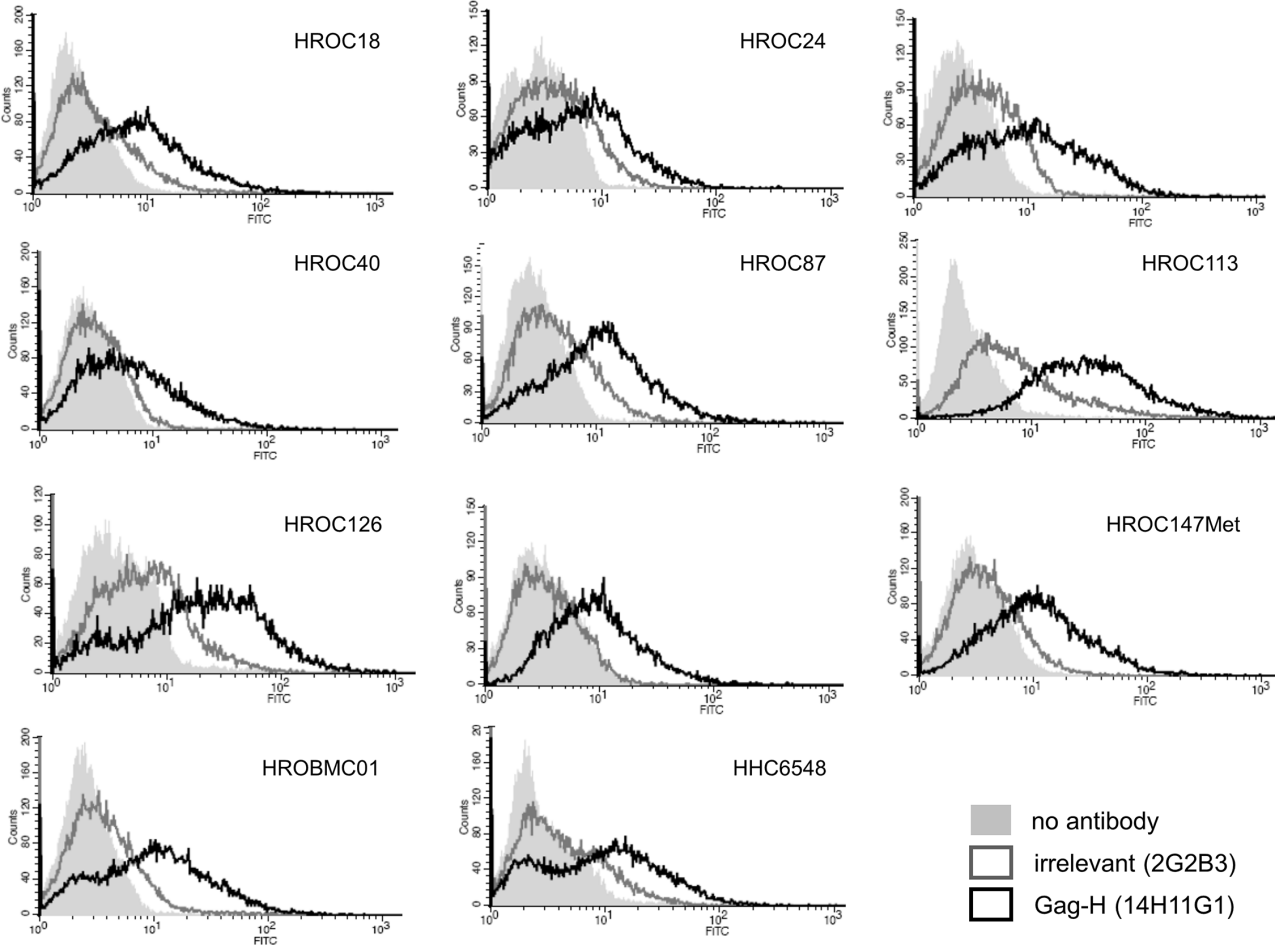
Gag-H protein expression with anti-Gag-H clone 14H11G1. Overlay of histograms obtained by flow cytometric analyses of 11 CRC cell lines with no primary antibody (filled light grey surface), irrelevant antibody (anti-His; 2G2B3; grey line) and anti-Gag-H antibody (clone 14H11G1; black line) are presented in the figure.

Expression of HERV-H Gag proteins was assessed by a flow cytometry screen of eleven low passage patient-derived CRC cell lines, established in our lab [24–26]. Peripheral blood mononuclear cells (PBMC) from three healthy donors, HEK293 cells and three low passage patient-derived glioma cell lines, also established in our lab [27,28], served as controls. Cells stained only with secondary antibody and cells stained with an irrelevant antibody served as antibody controls. The clone 14H11G1 was the most well performing one in all CRC cell lines analyzed (Fig 3 and S2 Fig), generally followed by 1B3H7, 1D7D11 and 2H2D6. CRC cell lines HROC18, HROC32, HROC87, HROC126, and HHC6548 as well as the brain metastatic cell line HROBMC01 generally showed the strongest staining. HROC147 derived from a primary CRC and HROC147Met established from its corresponding liver metastasis often showed marginal staining. HROC40 always had the weakest intensity (Fig 4). Virtually no expression was observed in PBMC from healthy donors, in glioma cell lines or human embryonic kidney cells (HEK293). Two out of the three B-lymphoid cell lines (B-LCL) from CRC patients also had no substantial staining; however one B-LCL was borderline positive with clone 14H11G1 (S3 Fig).

The HERV-H Gag protein expression of HROC cell lines was further analyzed by western blot using the clones 14H11G1, 1B3H7 and 1D7D11 in comparison to the irrelevant antibody. Respective 45kDa beta-actin control bands, analyzed as loading control, could easily be detected in all four blots. The approximate size of the recognized band for clone 14H11G1 is 58kDa, while the blots stained with irrelevant antibody or anti-Gag-H clones 1B3H7 and 1D7D11 show no bands at all (S4 Fig).

### Anti-Gag-H immunohistochemistry

**Fig 5:**
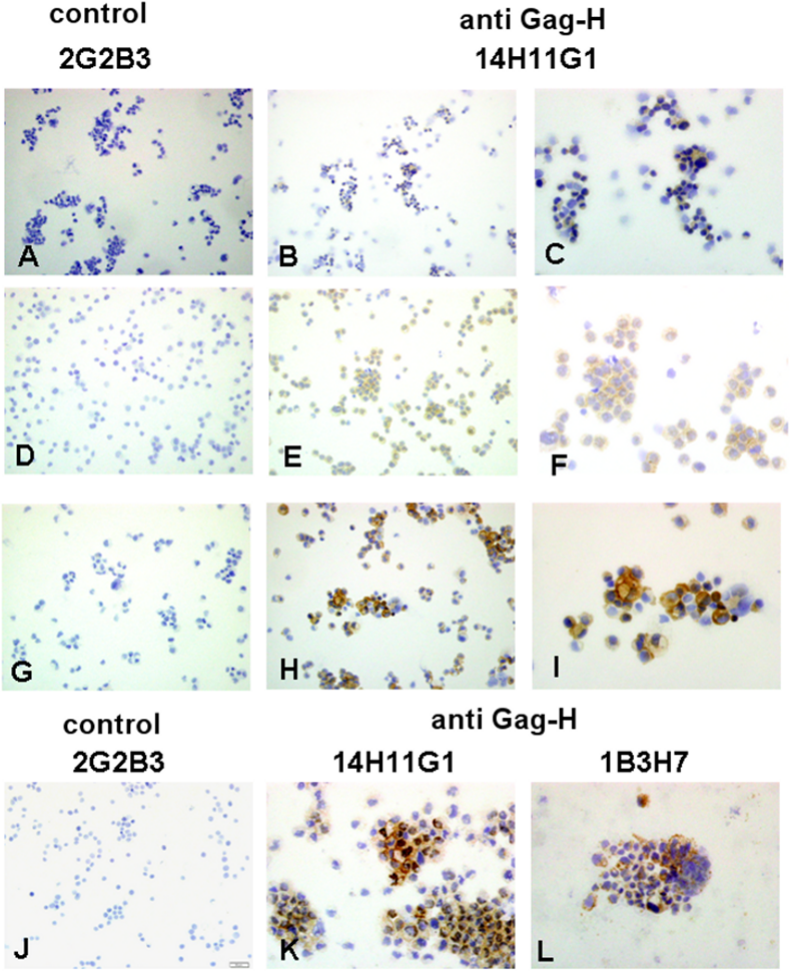
Examples of Gag-H immunohistochemistry in representative cytospins. Cytospins of HROC126 (A-C), HROBMC01 (D-F) and HROC32 (G-I) showed weak (HROC32) or strong (HROC126 and HROBMC01) cytoplasmic Gag-H expression (B, E, H by x20 objective; C, F, I by x40 objective) and no reaction with the control antibody 2G2B3 (A, D, G by 20x objective). Comparison of Gag-H expression of the two anti-Gag-H clones 14H11G1 and 1B3H7 in HROC147Met (J-L by x40 objective); no reaction with the control antibody 2G2B3 (K by 20x objective).

**Fig 6:**
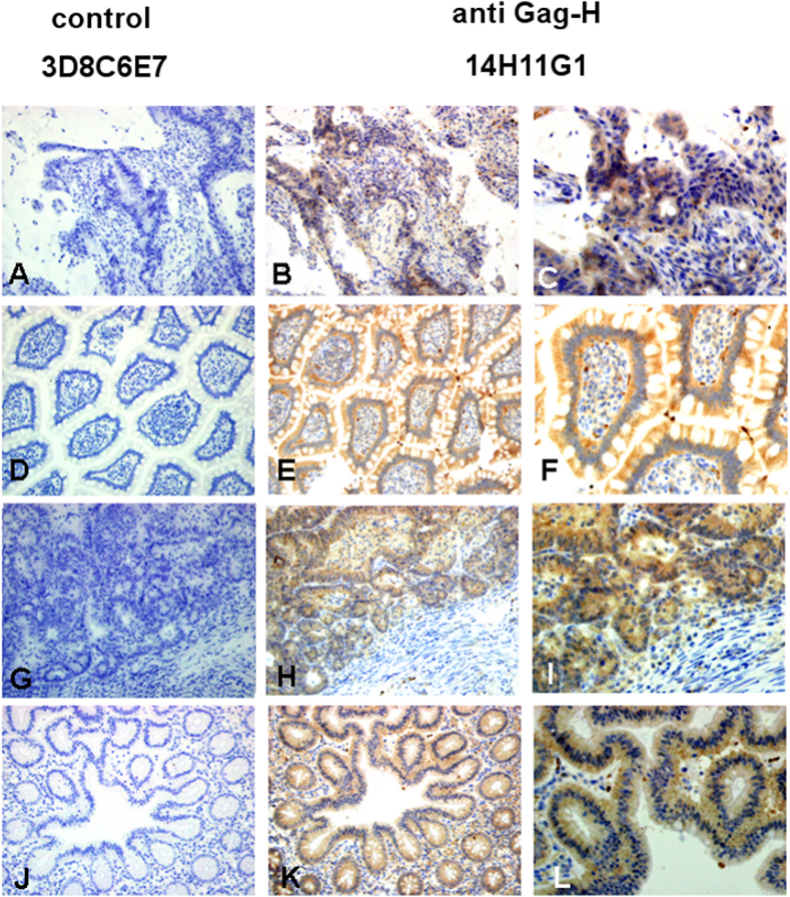
Examples of Gag-H immunohistochemistry in two representative clinical MSS cases. The upper row represents the tumor and the bottom row the normal tissue of the cases. Both cases: No. 6 (A-F) and No. 13 (G-L) showed strong cytoplasmic immunostaining of Gag-H in tumor and in normal tissue (B, E, H, K by x20 objective; C, F, I, L by x40 objective). Negative immunostaining was achieved with the control antibody 3D8C6E7 (A, D, G, and J by x20 objective).

**Fig 7:**
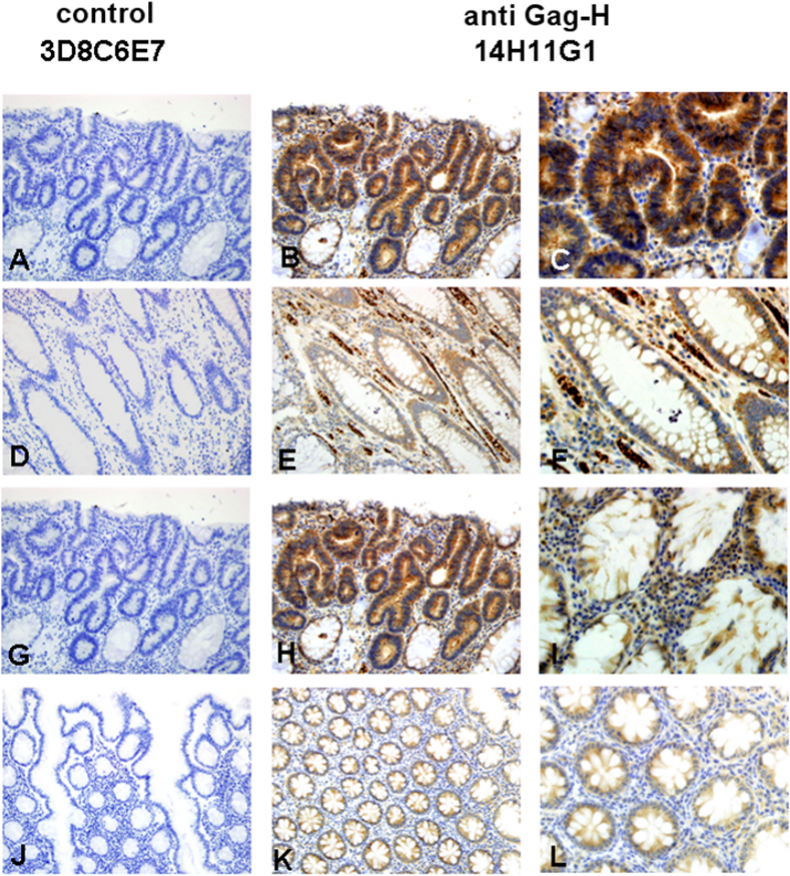
Examples of Gag-H immunohistochemistry in two representative clinical MSI cases. The upper row represents the tumor and the bottom row the normal tissue of the cases. Both cases: No. 8 (A-F) and No. 11 (G-L) showed strong cytoplasmic immunostaining of Gag-H in tumor and in normal tissue (B, E, H, K by x20 objective; C, F, I, L by x40 objective). Negative immunostaining was achieved with the control antibody 3D8C6E7 (A, D, G, and J by x20 objective).

For ensuing staining procedures, the anti-Gag-H antibody clones 14H11G1 and 1B3H7 were used in comparison to a control antibody. The immunohistochemical procedure was first established on cytospins of HROC cell lines (7 MSS cases, 5 MSI cases). In general, all cytospins exhibited no staining with the control antibody. Cytoplasmic immunostaining was noted in all seven cell lines tested with the clone 14H11G1, and in 7/11 lines tested with the clone 1B3H7 (Table 2, Fig 5). In the four cell lines staining negative with clone 1B3H7, staining with clone 14H11G1 was always positive (Table 2). In most cases, immunostaining was very heterogeneous with some completely unstained cells, others being weak and some even highly positive (Fig 5K). Such a diverse pattern makes unspecific staining with the two anti-Gag-H clones unlikely. At least in CRC cell lines, and for unknown reasons, Gag-H expression levels thus seem to vary. The established Gag-H immunohistochemistry with the clone 14H11G1 was transferred to clinical samples (paraffin sections from archived blocks). We analyzed 13 randomly selected pairs of CRC and matching normal tissue (7 MSS cases, 6 MSI cases). As with the cytospins, no immunostaining was detected with a control antibody. In detail, 11/13 tumors and 10/13 normal tissues exhibited moderate immunostaining with clone 14H11G1. Only weak immunostaining was noted in one tumor and 3 normal tissues; whereas one tumor was completely negative (Table 2). Occasionally, insular stronger staining was detected. In comparison to the results of the cytospins, diffuse immunostaining of the cytoplasm of tumor and normal crypt cells was noted. No obvious differences in the staining intensity between normal and tumoral cells were found. Furthermore, neither infiltrating lymphocytes, endothelial nor stromal cells gave positive staining signals. However, the immunostaining pattern in the FFPE tissue was different and stronger than the one noted in the cytospins of the CRC cell lines. Representative images of anti-Gag-H immunohistochemistry for the analyzed MSI and MSS cases are provided in Figs 6 and 7. Finally, we observed no significant differences between the cases of these two molecular classes.

**Table 2:** Summary of the immunohistochemistry results of selected CRC cases.

		molecular class	control	anti Gag-H clones	
				14H11G1	1B3H7
**Cytospins**					
	HROC113	MSI	-	+	-
	HROC69	MSS	-	weak	-
	HROBMC01	MSI	-	++	++
	HROC32	MSS	-	+	-
	HROC126	MSS	-	+	-
	HROC147Met	MSS	-	+	+
	HROC87 T0 M2	MSI	-	+	n.d.
	HHC6548	MSI	-	n.d.	weak
	HROC18	MSS	-	n.d.	+
	HROC40	MSS	-	n.d.	+
	HROC24	MSI	-	n.d.	+
	HROC147	MSS	-	n.d.	+
**FFPE tissue pairs**					
	#1 T	MSS	-	+	n.d.
	#1 N		-	+	n.d.
	#2 T	MSI	-	+	n.d.
	#2 N		-	weak	n.d.
	#3 T	MSS	-	-	n.d.
	#3 N		-	weak	n.d.
	#4 T	MSI	-	+	n.d.
	#4 N		-	weak	n.d.
	#5 T	MSI	-	+	n.d.
	#5 N		-	+	n.d.
	#6 T	MSS	-	+	n.d.
	#6 N		-	+	n.d.
	#7 T	MSS	-	weak	n.d.
	#7 N		-	+	n.d.
	#8 T	MSI	-	+	n.d.
	#8 N		-	+	n.d.
	#9 T	MSI	-	+	n.d.
	#9 N		-	+	n.d.
	#10 T	MSS	-	+	n.d.
	#10 N		-	+	n.d.
	#11 T	MSI	-	+	n.d.
	#11 N		-	+	n.d.
	#12 T	MSS	-	+	n.d.
	#12 N		-	+	n.d.
	#13 T	MSS	-	+	n.d.
	#13 N		-	+	n.d.

Control: antibodies 2G2B3 and 3D8C6E7; MSI, microsatellite instable; MSS, microsatellite stable; (+), moderate expression; (++), strong expression; (-), no expression; T, tumor; N, normal tissue; n.d., not done

## Discussion

Unique expression of several HERV-H loci in CRC samples (tissues and cell lines) was previously established [17–21]. Thus far, most analyses were solely based on RNA investigation either by RT-PCR experiments or custom designed HERV expression chip analyses. Only in one study, polyclonal anti-HERV-H Env antibodies have been generated and elevated HERV-H Env surface expression was demonstrated on B cells and monocytes of patients with active multiple sclerosis [29]. In the present study, monoclonal antibodies were generated against a Gag protein sequence of the HERV-H family to obtain a tool for HERV-H Gag protein analyses. The generated antibody clones seem to be (at least to a certain extent) pan-specific, since the identified epitopes map (with minimal variation) to multiple HERV-H gag loci in the human genome. The observed cytoplasmic Gag-H protein distribution is in accordance with published retroviral Gag protein localization [7,30]. The retroviral Gag proteins are generally synthesized as a poly-protein which is subsequently cleaved by the viral protease to produce the major structural proteins matrix, capsid and nucleocapsid [5,6]. Uncleaved Gag allows assembly and virus release, but the particles are immature, present a changed morphology and are not infectious [5]. However, unlike in murine models [31], in human CRC, production of virus-like-particles–either encoded by HERV or other viruses–is currently not supported by data from the literature. The observed band in western blot using the anti-Gag-H clone 14H11G1 is in size between the full-length and the intermediate product of porcine endogenous retrovirus Gag protein as recognized by a monoclonal antibody generated by Chiang and coworkers [30]. These data are somewhat conflicting. From the missing production of virus-like-particles, it seems safe to conclude that neither functional and cleaved Gag nor uncleaved Gag-proteins are produced in CRC. The western blot data of 14H11G1 imply that proteins with at least high similarity to Gag-H are present in the cytoplasm of colonic cells. It remains elusive if the anti-Gag-H antibodies recognize actual Gag-H-protein or epitopes of putative Gag-H fusion proteins. Unspecific reactivity of 14H11G1 could be excluded (I) due to the precise epitope mapping to Gag-H protein(s) and (II) the absence of cross-reactivity with other HERV families. The observed pan-colonic expression of HERV-H Gag is also in line with the results of Sacha and coworkers which found HERV-K Gag expressed in epithelial cells and glands of the small intestine [32].

So far, Syncytins, which are expressed in the context of syncytiotrophoblast generation by cellular fusion events in the human placenta [33,34], are the only HERV-derived proteins with physiological function. The precise nature / locus of colon-specifically expressed Gag-H (fusion) proteins will (with the help of 14H11G1) be the subject of future investigations aimed at unraveling the functional role of HERV-H-encoded proteins in the gut.

HERV-H has been shown to induce epithelial to mesenchymal transition, support metastasis and help recruit immunosuppressive immune cells to the tumor site [35]. This functionality was attributed to sequences and peptides encoded by *env* genes of HERV-H. Similar expression levels and pattern of Gag-H in normal colonocytes and in CRC cells raise doubts on a specific functional role of Gag-H proteins in CRC development or maintenance.

Another puzzling result was the borderline expression of Gag-H in one analyzed EBV-transformed B cell line (B-LCL). Different viral infections have been shown to positively influence HERV expression, as for example HERV-K activation by HIV [36] andactivation of the Syncytin-encoding and MS-associated HERV-W by EBV. Thus, it is possible that EBV used for immortalization of the peripheral blood B cells, is the causative agent for Gag-H expression here, too. However, in analogy to the observed expression of HERV-H Env protein in multiple sclerosis patients [29], the HERV-H Gag expression might also be due to the cancerous disease of the patient the B-LCL was derived from.

Although our analysis failed to demonstrate cancer-specificity for Gag-H expression, the anti-Gag-H antibodies may still be useful to determine colonic origin (e.g. in metastases of unknown origin and cancers of unknown primary) of metastatic cells. Moreover, the anti-Gag-H antibodies may be suited for screening of circulating tumor cells in blood, serum or urine samples. This would substantially add to the repertoire of non-invasive screening approaches for CRC.

Finally, the investigation of Gag-H protein expression in colon (normal and cancerous) epithelial cells may have turned up a number of questions as to the specific role for Gag-H proteins in the gut and a functional involvement in colonic epithelial cells. With the present study, the starting point for more detailed analyses has been set. The uniqueness of Gag-H protein expression for colon tissues (in parallel to the colonic and CRC restricted RNA expression from HERV-H loci [20,21]) can initially be addressed by qPCR and validated in IHC staining of tissue sections and complement our flow cytometry data on PBMC, kidney, glioma and B-LCL. A potential correlation with cell cycle state of Gag-H protein expression and an involvement of the protein in acquisition or maintenance of a particular cell state may also be investigated.

## Conclusion

This manuscript describes the generation of the first monoclonal antibodies directed towards Gag proteins of the HERV-H family. Initial characterization experiments revealed frequent cytoplasmic expression of Gag-H in CRC cell lines and clinical cases as well as in normal colonocytes. The latter is a rather surprising finding as it implies a potential physiological role of Gag-H in the normal gut.

## Supporting Information

S1 Fig Amino acid sequence of recombinant Gag-H protein. The figure depicts the amino acid sequence of the recombinant GST-Gag-H fusion protein used for antibody generation. The GST sequence is colored in blue, the Gag-H sequence in black and the His tag in red.
(PPT)

S2 Fig Gag-H protein expression in CRC cell lines. Expression of Gag-H proteins was assessed in eleven CRC cell lines using all four monoclonal mouse anti-Gag-H antibody clones. Mean fluorescence intensity (MFI) is depicted in the bar chart. Results represent the mean of three independent flow cytometry experiments and standard deviation.
(PPTX)

S3 Fig Gag-H expression in non-CRC cells (PBMC, HEK293, glioma and B-LCL). Expression of Gag-H proteins for PBMC of three healthy volunteers (A) three patient derived glioma cells (B) B-LCL of three CRC patients (C) and the human embryonic kidney cell line HEK293 (D) were analyzed. Expression of cells stained with irrelevant control antibody (anti-His, 3G3B2; upper row) and the most well performing anti-Gag-H antibody clone (14H11G1; lower row) are depicted in dot-plot and histogram charts.
(PPT)

S4 Fig Gag-H protein expression analyses by western blot. Expression of Gag-H proteins in CRC cell lines was assessed in western blot analyses using the anti-Gag-H antibody clones 14H11G1, 1B3H7 and 1D7D11 (red). Sufficient protein loading was verified using an anti-actin antibody (green). Approximate sizes are given.
(PPTX)

S1 Table Epitope mapping. (XLS)

S2 Table Bioinformatics analyses of Gag-H epitope distribution in the human genome. (XLS)

## Abbreviations

AA: amino acid
B-LCL: B lymphoid cell line
CRC: colorectal carcinoma
Gag-H: HERV-H gag protein
HERV: human endogenous retrovirus
MSI: microsatellite instability
MSS: microsatellite stable
PBMC: peripheral blood mononuclear cells
