# Supplementary material for: Generation, Characterization and Application of Antibodies Directed against HERV-H Gag Protein in Colorectal Samples
Source: PLoS One. 2016 Apr 27;11(4):e0153349. doi: 10.1371/journal.pone.0153349 (PMC4847760; doi:10.1371/journal.pone.0153349)
Supplement: S1 Fig — The figure depicts the amino acid sequence of the recombinant GST-Gag-H fusion protein used for antibody generation. The GST sequence is colored in blue, the Gag-H sequence in black and the His tag in red. (PPT) [file pone.0153349.s001.ppt]

## Slide 1
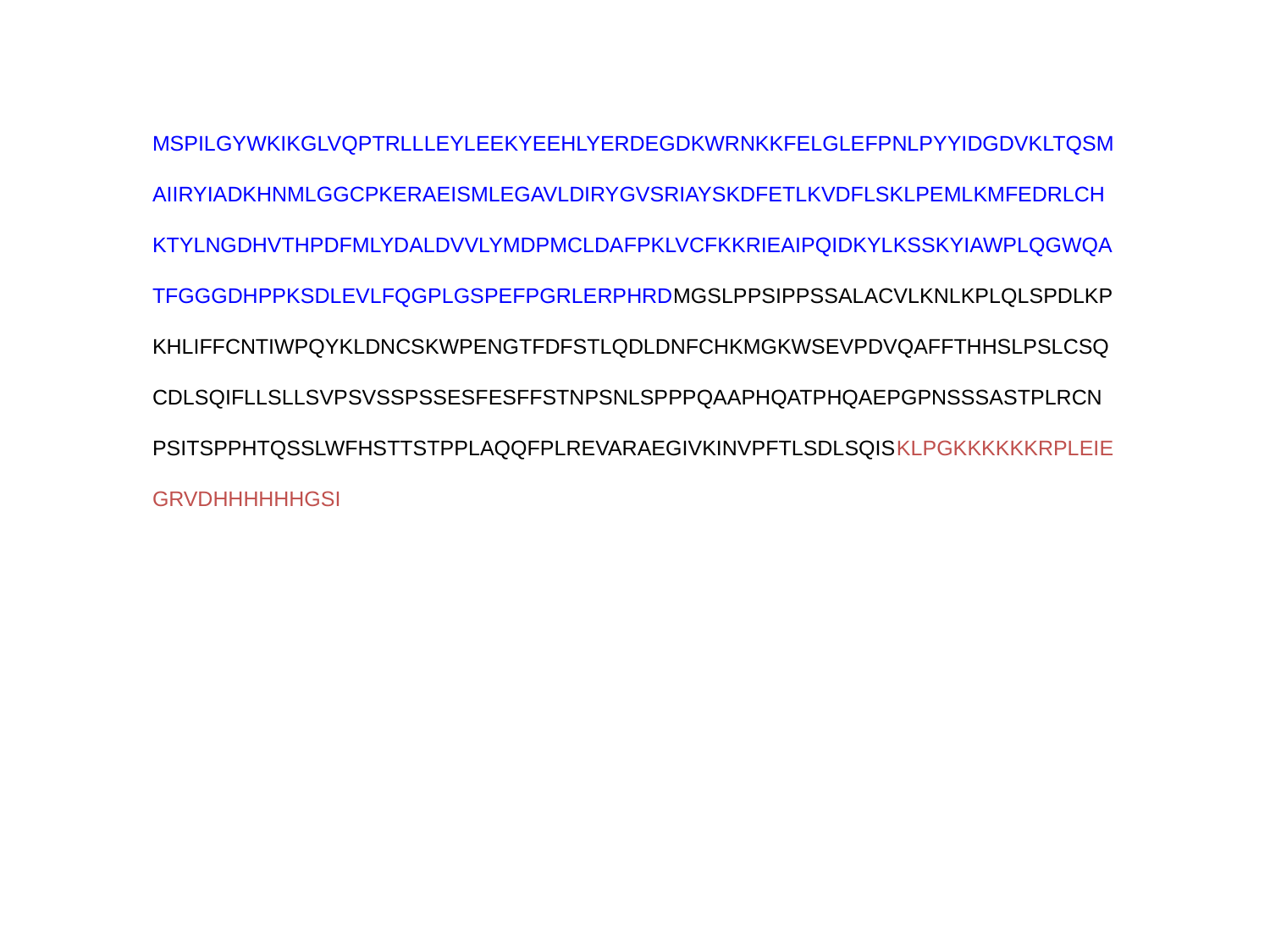

MSPILGYWKIKGLVQPTRLLLEYLEEKYEEHLYERDEGDKWRNKKFELGLEFPNLPYYIDGDVKLTQSMAIIRYIADKHNMLGGCPKERAEISMLEGAVLDIRYGVSRIAYSKDFETLKVDFLSKLPEMLKMFEDRLCHKTYLNGDHVTHPDFMLYDALDVVLYMDPMCLDAFPKLVCFKKRIEAIPQIDKYLKSSKYIAWPLQGWQATFGGGDHPPKSDLEVLFQGPLGSPEFPGRLERPHRDMGSLPPSIPPSSALACVLKNLKPLQLSPDLKPKHLIFFCNTIWPQYKLDNCSKWPENGTFDFSTLQDLDNFCHKMGKWSEVPDVQAFFTHHSLPSLCSQCDLSQIFLLSLLSVPSVSSPSSESFESFFSTNPSNLSPPPQAAPHQATPHQAEPGPNSSSASTPLRCNPSITSPPHTQSSLWFHSTTSTPPLAQQFPLREVARAEGIVKINVPFTLSDLSQISKLPGKKKKKKRPLEIEGRVDHHHHHHGSI
