# Supplementary figures and images for: Generation, Characterization and Application of Antibodies Directed against HERV-H Gag Protein in Colorectal Samples
Source: PLoS One. 2016 Apr 27;11(4):e0153349. doi: 10.1371/journal.pone.0153349 (PMC4847760; doi:10.1371/journal.pone.0153349)

## Slide 1
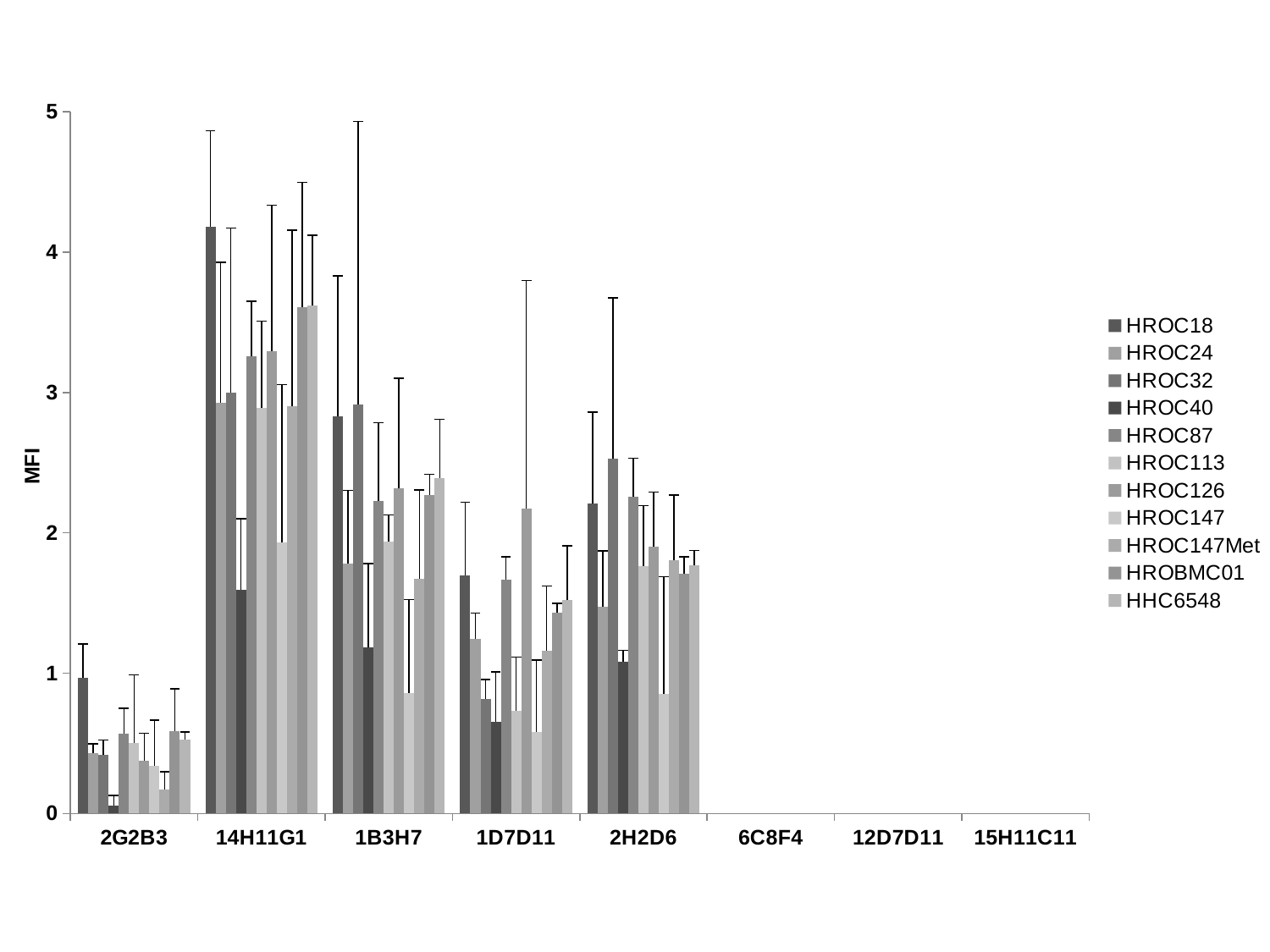

[unsupported chart]

Supplement: S2 Fig — Expression of Gag-H proteins was assessed in eleven CRC cell lines using all four monoclonal mouse anti-Gag-H antibody clones. Mean fluorescence intensity (MFI) is depicted in the bar chart. Results represent the mean of three independent flow cytometry experiments and standard deviation. (PPTX) [file pone.0153349.s002.pptx]

## Slide 1
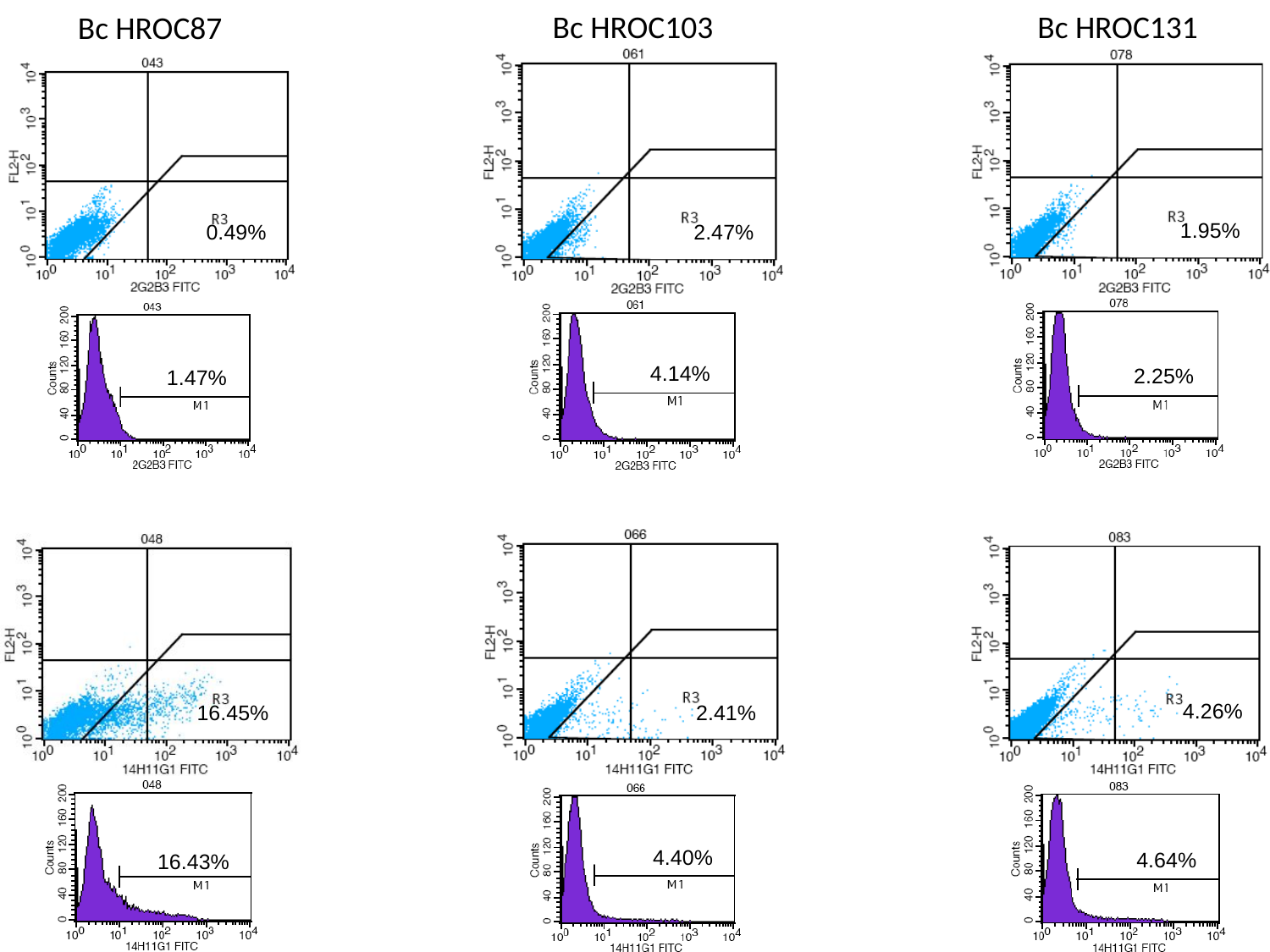

Bc HROC103
Bc HROC131
Bc HROC87
1.95%
0.49%
2.47%
4.14%
2.25%
1.47%
4.26%
16.45%
2.41%
4.40%
4.64%
16.43%

## Slide 2
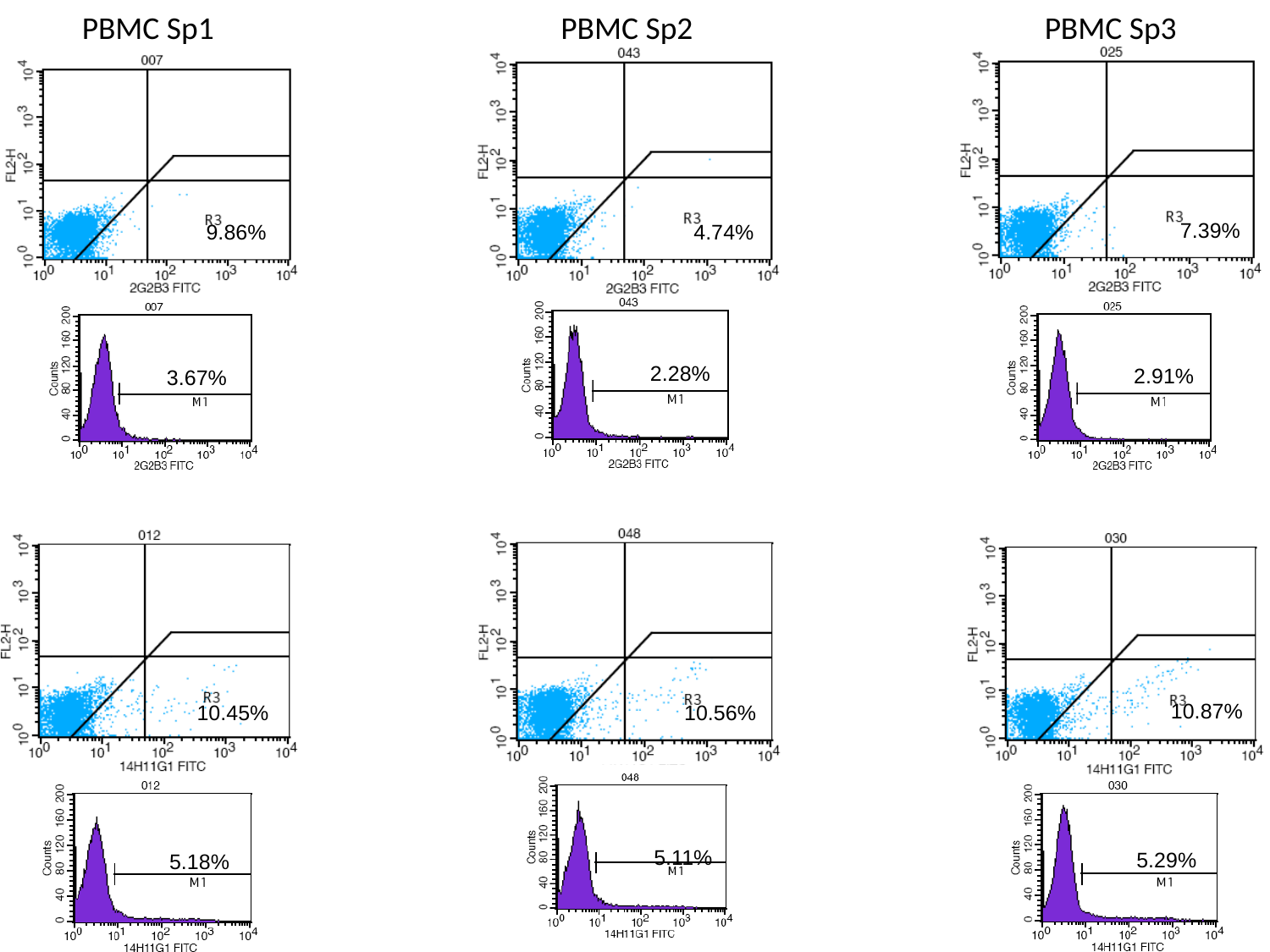

PBMC Sp1
PBMC Sp2
PBMC Sp3
7.39%
9.86%
4.74%
2.28%
2.91%
3.67%
10.87%
10.45%
10.56%
5.11%
5.29%
5.18%

## Slide 3
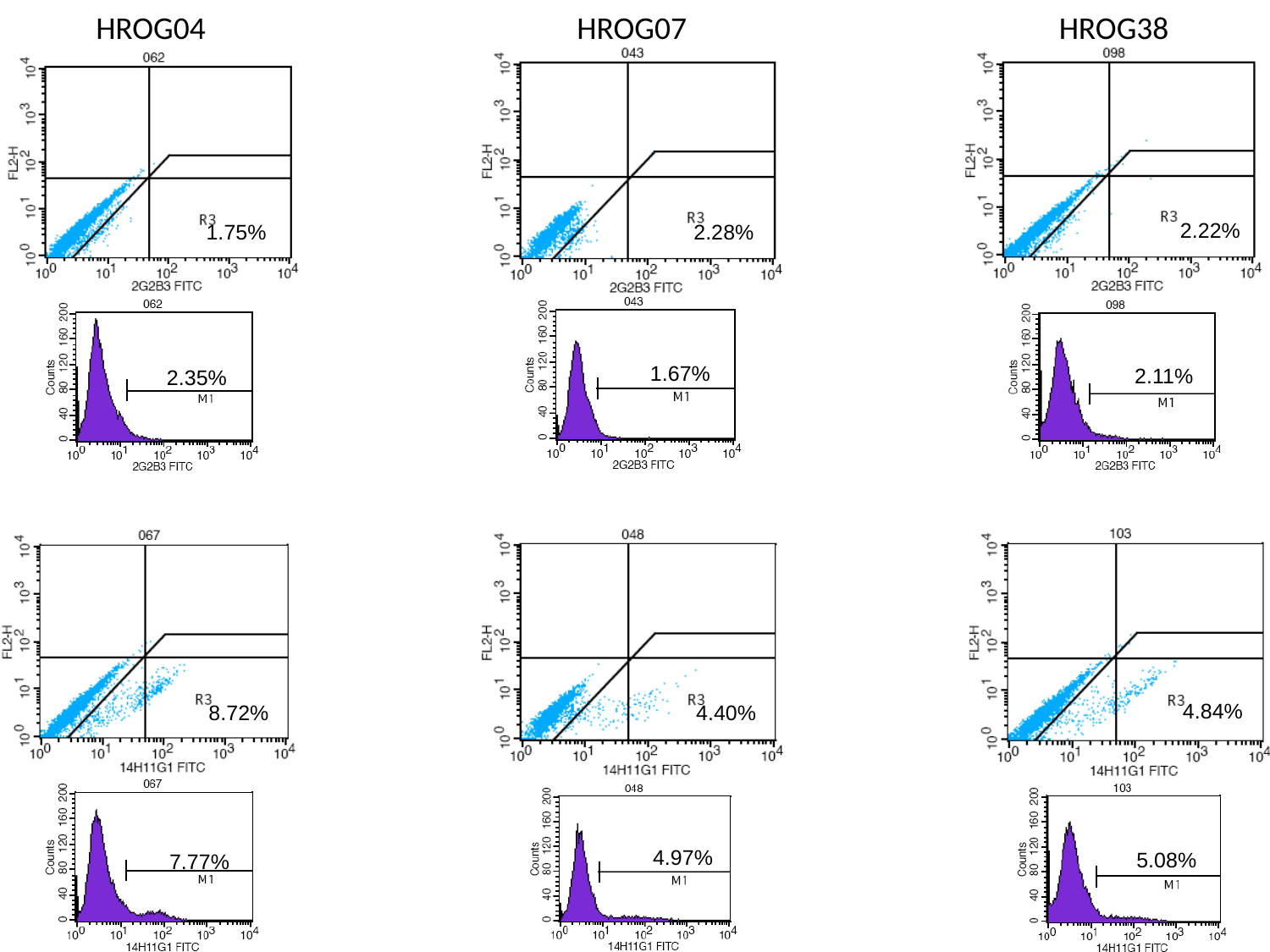

HROG04
HROG07
HROG38
2.22%
1.75%
2.28%
1.67%
2.11%
2.35%
4.84%
8.72%
4.40%
4.97%
5.08%
7.77%

## Slide 4
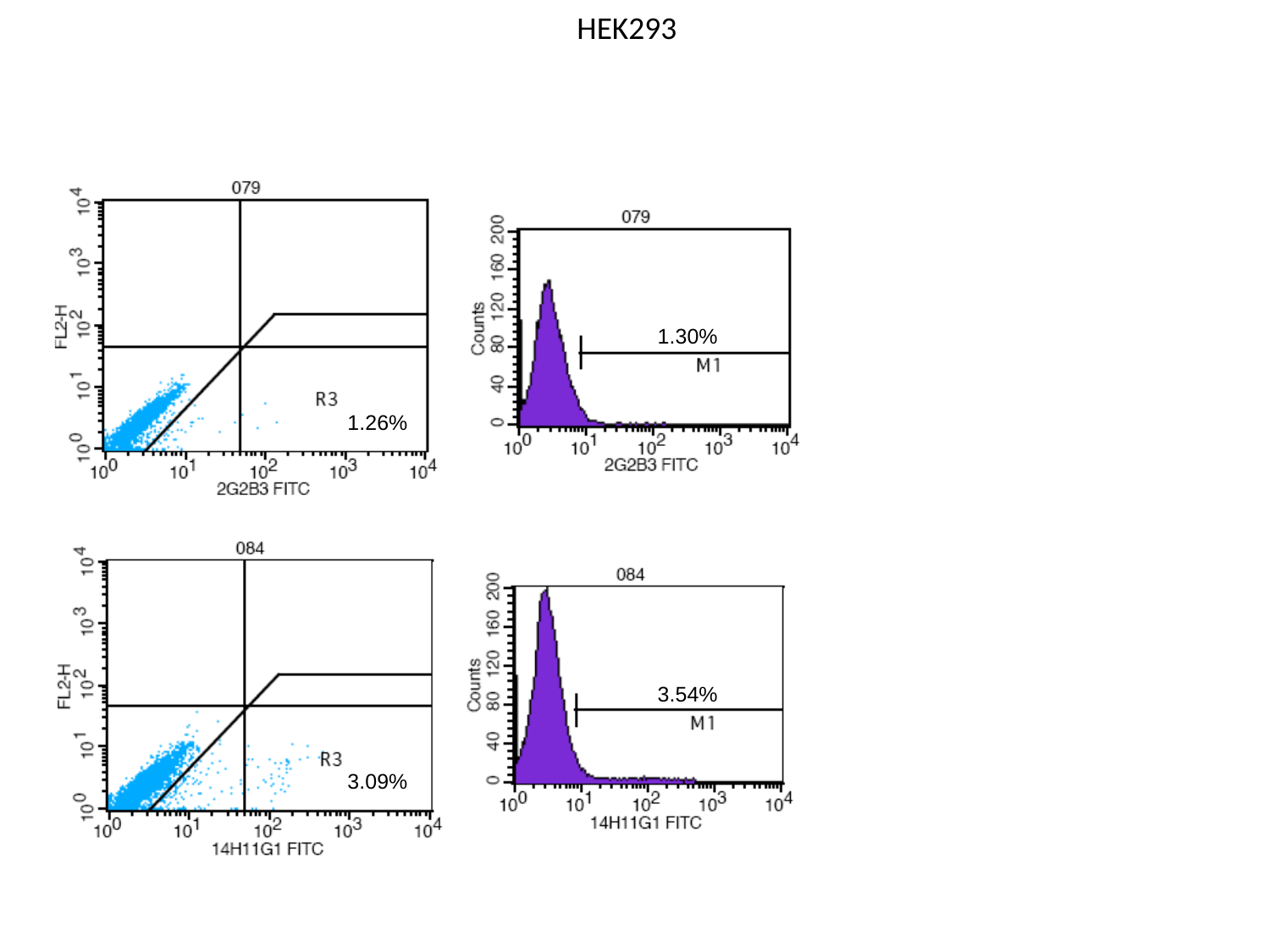

HEK293
1.30%
1.26%
3.54%
3.09%

Supplement: S3 Fig — Expression of Gag-H proteins for PBMC of three healthy volunteers (A) three patient derived glioma cells (B) B-LCL of three CRC patients (C) and the human embryonic kidney cell line HEK293 (D) were analyzed. Expression of cells stained with irrelevant control antibody (anti-His, 3G3B2; upper row) and the most well performing anti-Gag-H antibody clone (14H11G1; lower row) are depicted in dot-plot and histogram charts. (PPT) [file pone.0153349.s003.ppt]
