# Supplementary material for: Generation, Characterization and Application of Antibodies Directed against HERV-H Gag Protein in Colorectal Samples
Source: PLoS One. 2016 Apr 27;11(4):e0153349. doi: 10.1371/journal.pone.0153349 (PMC4847760; doi:10.1371/journal.pone.0153349)
Supplement: S4 Fig — Expression of Gag-H proteins in CRC cell lines was assessed in western blot analyses using the anti-Gag-H antibody clones 14H11G1, 1B3H7 and 1D7D11 (red). Sufficient protein loading was verified using an anti-actin antibody (green). Approximate sizes are given. (PPTX) [file pone.0153349.s004.pptx]

## Slide 1
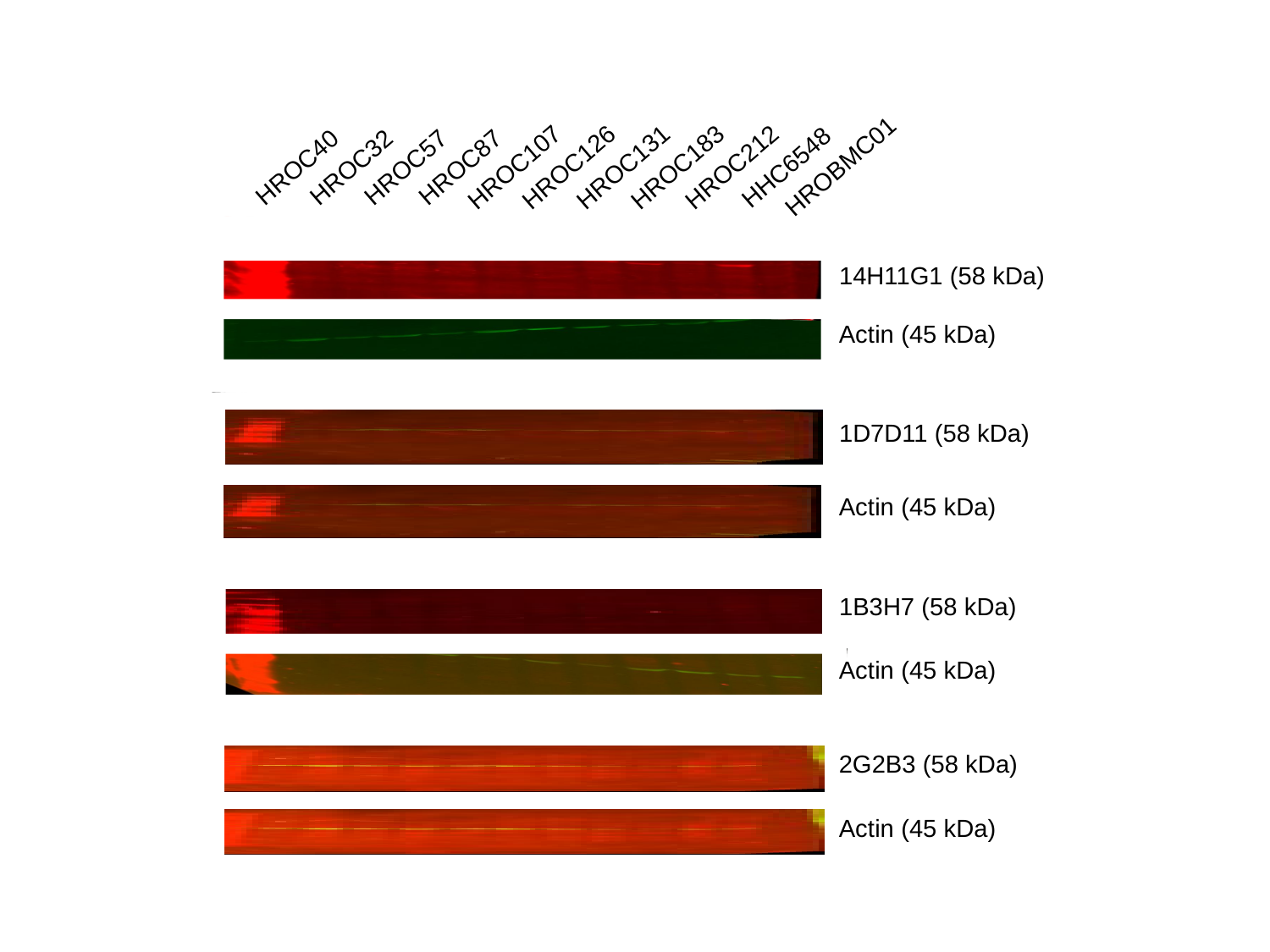

HROBMC01
HROC107
HROC126
HROC131
HROC183
HROC212
HROC40
HROC32
HROC57
HROC87
HHC6548
14H11G1 (58 kDa)
Actin (45 kDa)
1D7D11 (58 kDa)
Actin (45 kDa)
1B3H7 (58 kDa)
Actin (45 kDa)
2G2B3 (58 kDa)
Actin (45 kDa)
